# Comparing the case mix and survival of women receiving breast cancer care from one private provider with other London women with breast cancer: pilot data exchange and analyses

**DOI:** 10.1186/s12885-016-2439-2

**Published:** 2016-07-07

**Authors:** Elizabeth A. Davies, Victoria H. Coupland, Steve Dixon, Kefah Mokbel, Ruth H. Jack

**Affiliations:** Public Health England, Knowledge and Intelligence (London), 2nd Floor, Skipton House, 80 London Road, London, SE1 6HL UK; King’s College London, Health and Social Care Research, Faculty of Health Sciences & Medicine, 5th Floor, Capital House, 42 Weston Street, London, SE1 3QD UK; HCA International Ltd, 242 Marylebone Road, London, NW1 6JL UK; Health and Social Care Research, Faculty of Health Sciences & Medicine, 5th Floor, Addison House, London, SE1 IUL UK

**Keywords:** Breast cancer, Survival, Inequalities, Health care organisation

## Abstract

**Background:**

Data from providers of private cancer care are not yet formally included in English cancer registration data. This study aimed to test the exchange of breast cancer data from one Hospital Corporation of America International (HCAI) hospital in London with the cancer registration system and assess the suitability of these data for comparative analyses of case mix and adjusted survival.

**Methods:**

Data on 199 London women receiving ‘only HCAI care’, 278 women receiving ‘some HCAI care’ (HCAI and other services), and 31,234 other London women diagnosed between 2005 and 2011 could be identified and compared. Overall survival was estimated using the Kaplan-Meier method, and Cox regression was used to adjust for age, socioeconomic deprivation, year of diagnosis, stage of disease and recorded treatment.

**Results:**

Women receiving ‘only HCAI care’ were younger, lived in areas of higher affluence (47.8 % vs 27.6 %) and appeared less likely to be recorded as having screen-detected (2.5 % vs 25.0 %) disease than other London women. Women receiving ‘some HCAI care’ were more similar to ‘HCAI only’ women. Although HCAI stage of disease data completeness improved during the study period, this was less complete overall than cancer registration data and limited the comparative survival analyses. An apparent survival advantage for ‘HCAI only’ women compared with other London women (hazard ratio 0.48, 95 % confidence interval (CI): 0.32-0.74) was attenuated and no longer statistically significant after adjustment (0.79, 95 % CI: 0.51-1.21). Women receiving ‘some HCAI care’ appeared to have higher survival (hazard ratio 0.24, 95 % CI 0.14-0.41) which was attenuated to 0.48 (95 % CI: 0.28-0.80) in the fully adjusted model.

**Conclusions:**

Exchange of data between the private cancer sector and the English cancer registration service can identify patients who receive all or some private care. The better survival of women receiving only or some HCAI breast cancer care appears to be at least partly explained by demographic, disease, and treatment factors. However, larger studies using similarly quality assured datasets and more complete staging data from the private sector are needed to produce definitive comparative results.

## Background

Successive English health policies including the 2012 NHS Health and Social Care Act [1] have encouraged some private sector health care organisations to become interested in bidding for contracts to provide National Health Service (NHS) care. The proportion of English NHS spending delivered via non-NHS providers increased from 2.8 % over 2006 to 2007 to 5.9 % over 2013 to 2014 [2] while people holding some form of private health insurance declined from 12.5 % in 2006 to 10.9 % in 2012 [3]. However data on clinical outcomes for patients in the private sector remain sparse compared to those available for NHS care.

In the absence of comparative data the terms of engagement by which the private sector bid in competition with the NHS to provide a service remain controversial. Opponents have argued that private providers could opt to treat less complex cases and do not pay the full costs of medical complications or staff training, while some private providers have argued they are offered too few opportunities to bid and that payment does not reflect the full cost of service provision [4]. Other issues being debated include the potential benefits of private sector investment, innovation, and competition for the NHS, versus the risks posed by an uncertain health care market, new bureaucracy costs, a two tier system of services, and lack of strategic planning [5].

A key question in the debate about the provision of private services for the NHS must be whether the patients currently treated by private providers differ significantly in case mix, or have better outcomes than those treated by the NHS after these are taken into account. There are few published studies on this topic although analyses of data on NHS patients referred to independent treatment centres for routine surgical procedures suggest they are healthier, more affluent, and report better outcomes and fewer complications than similar patients treated at NHS hospitals [6–8]. While some private providers are now making information about patient experience, hospital acquired infection, and outcomes such as cardiac surgery survival available [9], detailed comparative information on the case mix and outcomes of patients treated for cancer is not yet available. This is because the systems for recording information on patients cared for in the private sector are less well-developed, have not been the focus of successive NHS policies for collection and quality assurance and private providers do not have access to the routinely available follow-up and death information collected for NHS patients. Private providers have also sometimes been excluded from national cancer audits despite a willingness to share their data and report outcomes [10]. The Private Healthcare Information Network (PHIN) is now developing with the aims of collecting much more information for patients and of providing comparative information on hospitals and consultants by 2017 [11].

Given the large numbers of people who can expect to develop cancer during their lifetime, the lack of information about outcomes for those who hold private insurance or choose to pay for private cancer care represents a gap in knowledge for English cancer policy. Historically data on some English patients receiving cancer care from private providers have been collected and held by the eight former regional cancer registries because private patients often receive pathology services or some part of their treatment in NHS hospitals. Registries have developed increasingly formal arrangements for collecting cancer data directly from private providers, but coverage has tended to be patchy. It has not therefore been possible to be sure that all privately diagnosed and treated patients have been included in population-based cancer registers. Information about these groups would be of significant interest in understanding existing national analyses, extending analyses of cancer inequalities and in making equitable policy decisions. For example, it is established that breast cancer patients living in affluent areas are more likely to have had their cancer diagnosed at an earlier stage of disease and to have a better survival [12–14]. The exclusion of patients treated privately from national and local analyses of cancer survival could, therefore, lead to an underestimate of current survival as well as in inequalities in survival between different socioeconomic groups. Data on London women whose breast cancer was detected by screening between 1999 and 2006 also suggested an under-representation of those in highest socioeconomic group whose cancers might have been detected by private screening services [15, 16].

To our knowledge no published UK studies have analysed data on cancer patients treated by private providers or compared their outcomes to those treated by NHS or other private services. We believe that access to data from private providers of a similar quality to those available for NHS would enable new analyses with the potential to benefit all patients. The aims of this pilot study were 1) to test a method of data extraction and exchange between one private provider - the Hospital Corporation of America International (HCAI) - and the National Cancer Registration Service (NCRS) in London and 2) to examine the feasibility of comparing the case mix and adjusted survival of women resident in London and cared for by one HCAI provider with other London women identified from cancer registration data and diagnosed and treated between 2005 and 2011. Our intention in this study was to move towards the provision of better national data from one private provider in order to build better evidence in this area rather than to influence NHS policies for private cancer care.

## Methods

### Data collection and processing

During the study period 2005 to 2011 cancer registration for the area of South East England including London, Kent, Surrey and Sussex was carried out by the former Thames Cancer Registry (TCR) at King’s College London. TCR received information about new cases of cancer largely from National Health Service (NHS) hospitals in the area and information on the deaths of residents from the Office for National Statistics via the NHS Central Register. Trained cancer registration officers extracted further demographic and tumour details, and information on whether patients had surgical, radiotherapy, chemotherapy and hormonal treatment within six months of their diagnosis recorded in their medical records. Data were quality assured as they were added to a central database and duplicate cases eliminated. Inpatient NHS Hospital Episode Statistics (HES) data including self-assigned ethnicity data were obtained from the NHS Information Centre each year and linked to the registration data.

During the study period TCR received some information on diagnoses or treatment within private health care providers, often where pathological diagnoses were undertaken for these providers by NHS hospitals, or where patients went on to receive NHS care. One experienced cancer registration officer had also liaised with private providers in London to collect additional data on new diagnoses. This study aimed to complete data collection for the cohort of patients seen at The Princess Grace hospital, an HCAI provider in central London, to a comparable standard for cases already within the registry dataset. This hospital was chosen because of the relatively large number of breast cancer patients seen and the quality of the historical data available. 46 % of patients receiving HCAI breast care in London in 2010 were seen at the Princess Grace Hospital.

The Princess Grace Hospital Breast Services, known as the London Breast Institute, is one hospital within the externally and internally quality assured and peer-reviewed ISO 9001:2008 accredited HCAI cancer network. Services provide breast screening annually from the age of 40, breast diagnostic services including mammography, ultrasound, breast MRI and PET CT. All patients undergo triple assessment, including vacuum assisted biopsy. Breast surgery and inpatient chemotherapy are provided on site, and patients attend the Leaders in Oncology Care clinic on Harley Street for day case chemotherapy and The Harley Street Clinic for radiotherapy (both within the HCAI cancer network). All patients are discussed at weekly multi-disciplinary team meetings and treatments are provided in accordance with national and international guidelines including NICE and the Association of Breast Surgery guidance.

The first phase of the study was to extract available data from the central HCAI patient administration systems for the 1033 women recorded as seen at the study hospital with a new diagnosis of breast cancer in the years 2005 to 2011. These data were passed securely to the Registry for initial matching against the registration dataset in January 2013. Where HCAI records were incomplete but the patient was known to the Registry, data were updated using the registration dataset, including whether the cancer was detected through NHS screening, and whether the patient was known to have died. This exercise revealed that 580 HCAI patients, 56 % of the complete cohort, had some data recorded on the TCR dataset, but that the remainder were not known to the Registry.

The second stage of new data collection was carried out by the cancer registration officer who had worked with private providers. This officer used the established registry methods to extract and code data from HCAI administrative, clinical, and results records. The coded data was added on site by HCAI data staff to a database designed for the study in consultation with the Registry. HCAI staff also followed up all patients actively with clinicians to determine if the patient was alive, or if not to confirm the date of death. Where an overall disease stage was missing, an experienced HCAI breast cancer clinician (KM) reviewed all extracted data to assign a Tumour, Node and Metastasis (TNM) stage.

In April 2013 the eight regional English cancer registries joined to become one single National Cancer Registration Service (NCRS) within Public Health England (PHE), the new executive agency for public health. In November 2013 the study dataset was passed securely back to the London Office of the NCRS. The dataset was traced using the NHS demographic service to determine whether patients had further NHS numbers or known dates of death. Date of diagnosis was found to be missing in 282 HCAI cases and updated where possible in March 2014 when further historical HCAI electronic pathology data became available. The dataset was then reduced to patients resident in London who had valid London postcodes. Those without valid postcodes were assumed to be non-London residents or other women who had attended from abroad, and were excluded. Patients were then assigned to a socioeconomic deprivation quintile based on their lower super output area of residence (areas of around 1500 individuals) using the income domain of the Indices of deprivation 2007 [17].

Data on female residents of London diagnosed with breast cancer during 2005 to 2011 were then extracted from the former TCR dataset to provide a study comparison sample. Three patient groups were defined for analysis, 1) women receiving ‘only HCAI care’ (representing those found only within the HCAI provider data in the study hospital, 2) women receiving ‘some HCAI care’ (representing those within both the HCAI provider and the TCR datasets), and 3) Other London women known only to TCR data (representing largely women receiving NHS care, but also a small proportion cared for by other London private providers). For those with information in both datasets, where there were inconsistent values for date of diagnosis, age, socioeconomic deprivation or stage, registry data were given priority, and where these were absent or not known in the registry data the HCAI value was included. Although we had information on whether each woman had a record of receiving any surgery, radiotherapy or chemotherapy, we did not have information on whether this was missing rather than not recorded. There was also insufficient detail available to reconstruct exactly what each treatment had been, or where and when it had been received.

This study was covered by section 251 of the Health and Social Care Act which enables the collection and analysis of data for cancer registration in the UK population. This provision is not mandated in the UK and does not currently apply directly to private hospital providers. Unlike NHS trusts, private hospitals are required to obtain explicit consent from patients for the transfer of their data for cancer registration purposes. Following legal advice a specific data agreement for the study was established at the outset between HCAI (trading as The Harley Street Clinic) and the former TCR to cover the extraction, transfer, matching, tracing, and analysis of data by registry staff in a similar way as for NHS data. The cancer registration officer (EN) also signed a new confidentiality agreement that allowed them to work with new data systems in one HCAI office. The final HCAI data were not processed onto the cancer registration system to undergo the usual quality assurance procedures that precede formal registration, but were maintained instead securely as a separate dataset for the study. The final analysis used anonymised data and ethical approval was not required.

### Data analysis

Patients who could not be verified as having a date of diagnosis between 2005 and 2011 or living in London, along with any registrations generated where the only information was from a death certificate were excluded. The earliest tumour diagnosed was included and all subsequent tumours excluded for patients who had multiple tumours diagnosed in the period study.

Data on the three groups of ‘HCAI only’, ‘some HCAI’, and TCR only women were compared in terms of proportions by age, deprivation of area of residence, year of diagnosis, stage of disease, ethnicity, screen-detection, recorded treatment and receptor status. We calculated and compared the overall survival of the three cohorts of patients from date of pathological diagnosis to date of death, or end of follow-up, determined as the end of December 2012. Overall survival curves were estimated using the Kaplan-Meier method and differences assessed using a log-rank test. Cox regression analyses were used to adjust sequentially for other variables after examining data completeness. The variables included were age, socioeconomic deprivation, year of diagnosis, stage of disease, and recorded treatment (any radiotherapy, surgery, chemotherapy or hormonal therapy), with tests for heterogeneity or trend excluding not known categories used where appropriate for each variable.

## Results

**Fig. 1:**
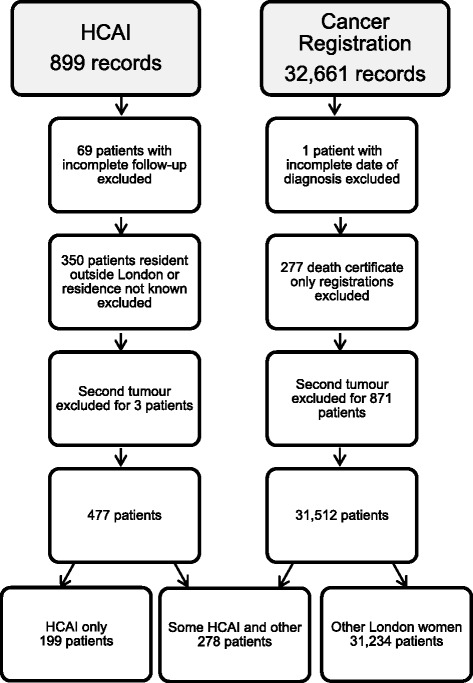
Data flow diagram for HCAI and cancer registration records included in the study

Figure 1 shows the data flow for the HCAI and TCR records and the removal of data for women not diagnosed in the study period, not resident in London, or with duplicate data or with registrations from a death certificate only. The final study sample therefore included 199 women known only to HCAI data, 278 known to both HCAI and TCR data, and 31,234 known only to TCR data.

Table 1 shows the demographic and clinical characteristics of these three study groups - 1) women receiving ‘HCAI care only’ 2) Women receiving ‘some HCAI care’ and 3) Other London women. The ‘HCAI care only’ group included a higher proportion of women aged under 50 than the other London women (49.2 % vs 24.9 %), and a lower proportion aged over 70 (12.5 % vs 28.8 %). The mean ages were 53 and 54 in the ‘HCAI care only’ and ‘some HCAI care’ groups, and 61 for other London women. The ‘HCAI care only’, and ‘some HCAI care’ groups also included a higher proportion of women living within areas in the two more affluent quintiles (47.8 % and 53.2 %, respectively) compared with 27.6 % in the other London women group.

**Table 1:** Demographic and clinical characteristics of women diagnosed with breast cancer 2005 to 2011 and receiving HCAI only care or some HCAI care compared with other London women

	HCAI care only		Some HCAI care		Other London women	
	*N*	%	*N*	%	*N*	%
Number of cases	199		278		31,234	
Mean age (years)	53		54		61	
Age group						
<50	98	49.2	131	47.1	7,792	24.9
50-59	33	16.6	56	20.1	7,243	23.2
60-69	43	21.6	58	20.9	7,210	23.1
70-79	17	8.5	18	6.5	4,783	15.3
80+	8	4.0	15	5.4	4,206	13.5
Socioeconomic deprivation quintile						
1 = Affluent	62	31.2	97	34.9	4,182	13.4
2	33	16.6	51	18.3	4,437	14.2
3	45	22.6	54	19.4	5,928	19.0
4	33	16.6	46	16.5	7,932	25.4
5 = Deprived	26	13.1	30	10.8	8,755	28.0
Year of diagnosis						
2005	17	8.5	22	7.9	4,286	13.7
2006	19	9.5	32	11.5	4,153	13.3
2007	51	25.6	37	13.3	4,366	14.0
2008	30	15.1	38	13.7	4,400	14.1
2009	29	14.6	57	20.5	4,649	14.9
2010	42	21.1	59	21.2	4,723	15.1
2011	11	5.5	33	11.9	4,657	14.9
Stage of disease						
1	64	32.2	109	39.2	9,836	31.5
2	42	21.1	95	34.2	10,311	33.0
3	1	0.5	28	10.1	2,979	9.5
4	2	1.0	6	2.2	2,639	8.4
Not known	90	45.2	40	14.4	5,469	17.5
Screening category						
Not Screen-detected	194	97.5	262	94.2	23,430	75.0
Screen-detected	5	2.5	16	5.8	7,804	25.0
Treatment						
Any cancer surgery	137	68.8	241	86.7	20,747	66.4
Any chemotherapy	61	30.7	140	50.4	9,841	31.5
Any radiotherapy	78	39.2	140	50.4	8,197	26.2
Any hormone therapy	40	20.1	121	43.5	8,964	28.7

Overall the proportion of women for whom stage of disease data was not known was higher in the ‘HCAI care only’ group (45.2 %) than in either the other London women (17.5 %) or the ‘some HCAI care’ group (14.4 %). However, the not known proportion decreased in the ‘HCAI care only’ group during the study period from 48.7 % in 2005 to 27.3 % for those diagnosed in 2011. Early stage disease (stage 1 and 2) was less frequent in women within the ‘HCAI care only’ group (53.3 %) than in either the other London women group (64.5 %) or the ‘some HCAI care’ group (73.4 %). However, when only patients with a known stage were included, 78 % of the other London women group had early stage disease, compared with 86 % of the ‘some HCAI care’ group and 97 % of the ‘HCAI care only’ group.

The proportion of women with screen-detected disease recorded within either the ‘HCAI care only’ (2.5 %) or ‘some HCAI care’ (5.8 %) groups was much lower than in the other London women group (25.0 %) The ‘some HCAI care’ group was more similar to the ‘HCAI care only’ group than to the other London women group for most characteristics except recorded treatment. Here higher proportions of the ‘some HCAI care’ group had surgery, radiotherapy, chemotherapy and hormone therapy recorded compared with the other two groups. Ethnicity was much less well-recorded in the HCAI data with 70.2 % (726/1033) of cases having incomplete data, while hormone receptor status was poorly recorded in each of the HCAI and the registration datasets for this period. These two variables could therefore not be considered further in the analysis.

**Fig. 2:**
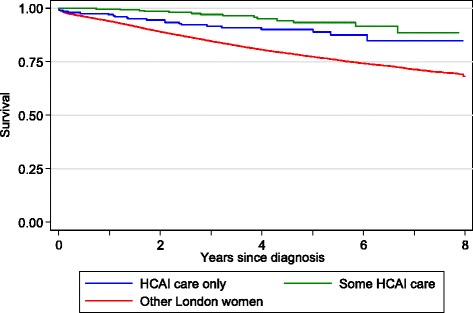
Kaplan-Meier survival estimates for women with breast cancer receiving HCAI only care, some HCAI care compared with other London women

Figure 2 shows the Kaplan-Meier overall survival curves for the three groups of women. The ‘HCAI care only’ patients had a better overall survival than the other London women, while the ‘some HCAI care’ patients had a better survival still (log-rank p < 0.0001). Table 2 shows the Cox proportional regression analysis results. The unadjusted hazard ratio (relative risk of mortality) for ‘HCAI care only’ patients compared with other London women was 0.48 (95 % confidence interval (CI): 0.32-0.74). After adjustment for age this survival advantage attenuated substantially to 0.66 (95 % CI 0.43-1.02) and was no longer statistically significant at the 5 % level, and after adjustment for deprivation was 0.70 (95 % CI 0.46-1.08). Year of diagnosis made little difference, while further adjustment for stage of disease increased the risk to 0.72 (95 % CI 0.47-1.10), and treatment increased it further to 0.79 (95 % CI 0.51-1.21). These results indicate that the main identified driver of the lower risk of mortality in the ‘HCAI care only’ women was age, with deprivation and treatment having influential though lesser effects. The unadjusted hazard ratio for the ‘some HCAI care’ patients was 0.24 (95 % CI 0.14-0.41) and was increased in a similar way to 0.48 (95 % CI 0.28-0.80) in the fully adjusted model. Age and treatment were the most influential drivers of lower risk of mortality among this group. Deprivation had a similar effect as in the ‘HCAI care only’ group.

**Table 2:** Mortality hazard ratios for women diagnosed with breast cancer between 2005 and 2011 and receiving HCAI care only and some HCAI care compared with other London women

	Unadjusted	Adjusted for age	Adjusted for age and deprivation	Adjusted for age, deprivation, and year of diagnosis	Adjusted for age, deprivation, year of diagnosis, and stage	Adjusted for age, deprivation, year of diagnosis, stage, and treatment
	HR (95 % CI)	HR (95 % CI)	HR (95 % CI)	HR (95 % CI)	HR (95 % CI)	HR (95 % CI)
Other London women	1.00	1.00	1.00	1.00	1.00	1.00
HCAI care only	0.48 (0.32, 0.74)	0.66 (0.43, 1.02)	0.70 (0.46, 1.08)	0.70 (0.45, 1.07)	0.72 (0.47, 1.10)	0.79 (0.51, 1.21)
Some HCAI care	0.24 (0.14, 0.41)	0.32 (0.19, 0.54)	0.34 (0.20, 0.58)	0.35 (0.21, 0.59)	0.40 (0.24, 0.67)	0.48 (0.28, 0.80)
*χ* ^2^-test (2 d.f.)	39.35	21.69	18.40	18.30	14.03	8.83
p-heterogeneity	<0.0001	<0.0001	<0.0001	0.0001	0.0009	0.0121
Age group						
<40		1.00	1.00	1.00	1.00	1.00
40-44		0.85 (0.72, 1.01)	0.86 (0.73, 1.02)	0.86 (0.73, 1.02)	0.88 (0.74, 1.03)	0.96 (0.81, 1.13)
45-49		0.77 (0.65, 0.89)	0.78 (0.67, 0.91)	0.78 (0.67, 0.92)	0.81 (0.70, 0.95)	0.89 (0.76, 1.04)
50-54		0.65 (0.56, 0.77)	0.67 (0.57, 0.78)	0.67 (0.57, 0.78)	0.78 (0.66, 0.91)	0.86 (0.74, 1.01)
55-59		0.83 (0.72, 0.97)	0.86 (0.74, 1.00)	0.85 (0.74, 0.99)	0.97 (0.84, 1.13)	1.11 (0.95, 1.29)
60-64		0.85 (0.73, 0.99)	0.88 (0.76, 1.02)	0.88 (0.76, 1.02)	1.06 (0.91, 1.23)	1.27 (1.10, 1.48)
65-69		1.05 (0.91, 1.21)	1.08 (0.93, 1.25)	1.08 (0.93, 1.24)	1.30 (1.12, 1.50)	1.65 (1.42, 1.91)
70-74		2.01 (1.74, 2.30)	2.07 (1.80, 2.39)	2.07 (1.80, 2.38)	2.24 (1.95, 2.58)	2.83 (2.45, 3.27)
75-79		2.85 (2.49, 3.26)	2.94 (2.57, 3.36)	2.94 (2.57, 3.36)	3.08 (2.69, 3.53)	3.91 (3.40, 4.50)
80+		6.19 (5.48, 6.99)	6.41 (5.67, 7.24)	6.42 (5.69, 7.26)	6.10 (5.40, 6.89)	6.87 (6.03, 7.82)
*χ* ^2^-test (1 d.f.)		2,755.90	2,804.15	2,804.01	2,540.64	2,052.19
p-trend		<0.0001	<0.0001	<0.0001	<0.0001	<0.0001
Socioeconomic deprivation quintile						
1 = Affluent			1.00	1.00	1.00	1.00
2			0.97 (0.88, 1.07)	0.96 (0.87, 1.06)	0.97 (0.87, 1.07)	0.97 (0.87, 1.07)
3			1.06 (0.96, 1.16)	1.05 (0.96, 1.15)	1.04 (0.95, 1.15)	1.04 (0.95, 1.14)
4			1.18 (1.08, 1.29)	1.18 (1.08, 1.29)	1.19 (1.09, 1.30)	1.16 (1.07, 1.27)
5 = Deprived			1.37 (1.26, 1.49)	1.36 (1.25, 1.48)	1.27 (1.17, 1.38)	1.22 (1.12, 1.33)
*χ* ^2^-test (1 d.f.)			92.81	92.14	61.05	44.50
p-trend			<0.0001	<0.0001	<0.0001	<0.0001
Year of diagnosis						
2005				1.00	1.00	1.00
2006				0.99 (0.91, 1.07)	1.08 (0.99, 1.17)	1.08 (1.00, 1.17)
2007				1.00 (0.92, 1.09)	1.10 (1.02, 1.20)	1.07 (0.98, 1.16)
2008				0.98 (0.89, 1.06)	1.11 (1.01, 1.21)	1.02 (0.93, 1.11)
2009				0.87 (0.79, 0.96)	0.93 (0.85, 1.03)	0.87 (0.80, 0.96)
2010				0.81 (0.73, 0.90)	0.82 (0.74, 0.91)	0.84 (0.76, 0.93)
2011				0.74 (0.65, 0.84)	0.81 (0.72, 0.92)	0.83 (0.73, 0.94)
*χ* ^2^-test (1 d.f.)				33.22	22.52	27.02
p-trend				<0.0001	<0.0001	<0.0001
Stage of disease						
1					1.00	1.00
2					1.02 (0.94, 1.11)	1.10 (1.01, 1.19)
3					2.28 (2.07, 2.51)	2.27 (2.06, 2.51)
4					6.37 (5.89, 6.90)	4.90 (4.52, 5.31)
Not known					2.67 (2.47, 2.88)	1.82 (1.68, 1.96)
*χ* ^2^-test (1 d.f.)					2,924.02	1,630.20
Excl. NK p-trend					<0.0001	<0.0001
Radiotherapy						
No						1.00
Yes						0.82 (0.77, 0.89)
*χ* ^2^-test (1 d.f.)						28.19
p-heterogeneity						<0.0001
Chemotherapy						
No						1.00
Yes						1.29 (1.20, 1.38)
*χ* ^2^-test (1 d.f.)						50.87
p-heterogeneity						<0.0001
Cancer surgery						
No						1.00
Yes						0.40 (0.37, 0.42)
*χ* ^2^-test (1 d.f.)						962.80
p-heterogeneity						<0.0001
Hormone therapy						
No						1.00
Yes						0.80 (0.75, 0.85)
*χ* ^2^-test (1 d.f.)						58.07
p-heterogeneity						<0.0001

## Discussion

### Summary of main findings

This study tested for the first time the retrospective exchange and transfer of breast cancer data for 2005 to 2011 between one private cancer care provider and the cancer registration service for London. While the exchange exercise was possible, the sample size was relatively small and we found that lack of comparable data for some key variables in the private provider data limited the conclusions we could draw from the analyses. We found that women receiving all or some of their care within one HCAI provider were often younger, living within areas of higher affluence, and less likely to be recorded as having screen-detected disease than other London women. However, disease stage, which is a key prognostic variable was less completely recorded within HCAI data than in cancer registration data, and HCAI women less often had early stage disease recorded than other London women. Using the available data on stage we found that women within the HCAI data appeared to have a better survival from their breast cancer than other London women, which was partly explained by their differing age, deprivation and recorded treatment. Data on ethnicity, tumour receptor status, and some aspects of specific treatment pathways were not available and could not be considered as explanatory variables.

### Limitations of the study

This is the first time that a large scale retrospective data exchange and transfer has been attempted between a private cancer provider and the cancer registration service for London where a significant proportion of private providers operate. As expected this was a complex undertaking and we found some problems emerging which limited the comparison of case mix and outcome that we could make. These included a wide catchment area for HCAI patients that substantially reduced the study sample of London residents, and differences in data collection and recording between the two systems despite similar data extraction techniques being used to create the new dataset.

To our knowledge there are no other published studies of case mix or outcomes for private cancer providers in the UK with which to compare our provisional findings. A significant difficulty for this study was the differential lack of high quality historical private sector data for key variables of a comparable quality to NHS data. Data on disease stage data were incomplete, though improving within the HCAI records and this limited the adequacy of the comparison and case mix adjustment that we could undertake in this analysis. It is possible, for example, that the lower proportion of early stage disease in the ‘HCAI only’ women was simply an artefact of the less complete recording of stage within HCAI data. To assess this possibility we undertook a series of sensitivity analyses. We carried out a complete case analysis and also used multiple imputation (20 imputations based on care group, age, deprivation, year of diagnosis, whether the cancer was screen-detected, treatment received, length of survival, and whether the patient was alive at the end of the study period). The complete case analysis increased the hazard ratios (fully adjusted ‘HCAI care only’: 1.28, 95 % CI: 0.71-2.32, ‘some HCAI care’: 0.63, 95 % CI 0.35-1.11, so that there was no statistically significant difference in the survival of all three groups. Multiple imputation gave a similar stage distribution in the two HCAI care groups (‘HCAI care only’: 44 % stage 1, 40 % stage 2, 10 % stage 3, 7 % stage 4; ‘some HCAI care’: 45 % stage 1, 39 % stage 2, 9 % stage 3, 7 % stage 4). There were 32 % of other London women imputed as having stage 1 disease, 41 % with stage 2, 14 % with stage 3, and 13 % with stage 4. The fully adjusted results using these imputed stage values were similar to the original results, particularly for the ‘some HCAI care’ group (0.40, 95 % CI: 0.29-0.68), though attenuated for the ‘HCAI care only’ group (0.89, 95 % CI: 0.57-1.38).

We also calculated 5-year net survival for the three groups, which takes background mortality into account, but not the differences in case mix and other factors between the groups. This was lowest for the other London women (84.1 %, 95 % CI: 83.4 %-84.8 %), higher for the ‘HCAI care only’ (93.6 %, 95 % CI: 86.8 %-97.0 %) and highest in the ‘some HCAI care’ group (97.3 %, 95 % CI: 86.7 %-99.5 %).

We also did not have complete data on other tumour factors or detailed information on treatment pathways that could have influenced outcome. It would have been preferable to have been able to subject these data to the same quality assurance and querying processes from the outset including for pathological diagnosis as occurs for data received for cancer registration from NHS hospitals in England. Indeed the fact that cancer registration is not mandated for cases diagnosed or treated in the private sector in the UK, unlike the situation for other countries means that this lever for quality improvement of the data is not yet available. Overall it also seems likely that some treatment data was missing in all three study groups. We would expect these data to be improving in completeness in more recent years. Screening information may also have been incomplete for those women who only received HCAI care and were diagnosed through the NHS screening programme. For this reason we did not include the screen-detected variable in the survival analyses. However, when we included the screening information in the fully adjusted model the conclusions remained the same, although the hazard ratios decreased to 0.69 (95 % CI 0.45-1.07) for the ‘HCAI care only’ group, and 0.43 (0.26-0.73) for the ‘some HCAI care’ group.

### Implications for policy and practice

We hope that this feasibility study will inform debate about the evidence for the private sector providing services to NHS patients by showing how cancer registration data could be collected from this sector and used to provide new information for the NHS, private providers, policy makers and the public. Collecting data from private providers of cancer services is one goal of the National Cancer Registration and Analysis Service within PHE [18]. Although English public health datasets aim to provide comprehensive coverage, in practice they exclude a small proportion of the population who opt to pay for private insurance, private healthcare services, or have these provided for them by their employers. For example, HES data, which are the main source of ethnicity data for many analyses of English hospital care, do not include data on all admissions to private hospitals.

Despite the long-standing interest of NHS and some private providers in discussing more frequent data exchange to inform the monitoring of commissioning decisions [19], there seem to have been barriers to overcome in making these practices routine. The consequence is a number of unanswered questions, particularly for cancer patients in London, about the uptake of cancer screening, the presentation of disease in different population groups and their outcomes. Some of these may be answered as HCAI and other private providers improve data collection within their systems towards NHS standards to allow further retrospective comparison. Other initiatives by the Care Quality Commission are also seeking to increase the information available about care within the independent sector [20]. If contracts with private providers are to become a part of the long-term provision for the NHS, they will need to be monitored and evaluated in a similar way to NHS services. One option to help improve cancer data quality in the private health sector would be the make cancer registration routine or mandatory in the same way as in other countries. While this would likely require political, business, legal and public debate the benefits of full population coverage would be significant. The findings of our study further suggest that comparing outcomes may be complex, particularly where patients move between providers in different sectors. In addition part of the better outcome for private providers suggested here may be explained by characteristics of women who select HCAI care or are selected into this care by their insurance policies. This would not be the situation for any future NHS service contracts where a representative section of London women might be expected to receive treatment in private sector hospitals.

### Implications for future research

Our study opens the way for larger comparative studies of breast cancer and other cancers treated across all HCAI providers in London, and by other private health care providers across the UK. Particular questions to be answered in addition to the issue of comparable stage data include whether there are differences in the ethnicity or tumour receptor status of patients receiving treatment by HCAI, whether delays in diagnosis, the treatments patients receive or the time they wait for them differs, and how women currently move between private and NHS care. The finding that women receiving 'some HCAI care' appeared to have better outcomes than the other two groups is intriguing but could clearly be a singular finding and requires replication in further larger datasets. One hypothesis is that it represents other unmeasured behavioural factors including women’s ability to negotiate two health sectors and receive several opinions on their treatment. For example, women with private insurance who have breast cancer detected at NHS screening could opt for initial private surgery and return to the NHS for later treatment when their insurance funds are depleted. In this respect the low rate of screen-detected disease in women receiving HCAI care was surprising given that our previous study of London NHS screening data had led us to suggest that women in the most affluent group may have been more likely to be diagnosed privately because they had lower screening uptake in some areas of London [17]. The findings in this new study could be a feature of the women attending this particular HCAI hospital or reflect the fact that this information is not recorded within HCAI data because insurance companies do not often support screening. Another unexpected finding was that patients who received HCAI and other care had more complete data for both stage of disease and treatment. This could be a consequence of better recording of data within the NHS, or could represent another singular finding from this relatively small study in one geographical area. A complete population-based view of London women being diagnosed and receiving treatment for breast cancer from all providers would therefore produce a more accurate picture of inequalities in outcome between different socioeconomic groups. We would hope that this will show where data is still needed to make a judgement about the size of and contributors to these inequalities, and possibly indicate new opportunities for reducing them.

## Conclusions

Exchange of data between the private cancer sector and the English cancer registration service can identify patients who receive all or some private care. The better survival of women receiving only or some HCAI breast cancer care appears to be at least partly explained by demographic, disease, and treatment factors. However, larger studies using similarly quality assured datasets and more complete staging data from the private sector are needed to produce definitive comparative results.

## Abbreviations

HCAI, Hospital Corporation of America International; HES, Hospital Episode Statistics; MRI, Magnetic Resonance Imaging; NCRAS, National Cancer Registration and Analysis Service; NCRS, National Cancer Registration Service; NHS, National Health Service; NICE, National Institute for Health and Care Excellence; PETCT, Positron Emission Tomography Computed Tomography; PHE, Public Health England; TCR, Thames Cancer Registry; TNM, Tumour Node Metastases; UK, United Kingdom.
